# Negligible Correlation between Radiographic Measurements and Clinical Outcomes in Patients Following Primary Reverse Total Shoulder Arthroplasty

**DOI:** 10.3390/jcm10040809

**Published:** 2021-02-17

**Authors:** Daniel P. Berthold, Daichi Morikawa, Lukas N. Muench, Joshua B. Baldino, Mark P. Cote, R. Alexander Creighton, Patrick J. Denard, Reuben Gobezie, Evan Lederman, Anthony A. Romeo, Knut Beitzel, Augustus D. Mazzocca

**Affiliations:** 1 Department of Orthopaedic Surgery, University of Connecticut, Farmington, CT 06030, USA; idarimo@hotmail.com (D.M.); lukas.muench@mri.tum.de (L.N.M.); baldino@uchc.edu (J.B.B.); mcote@uchc.edu (M.P.C.); mazzocca@uchc.edu (A.D.M.); 2 Department of Orthopaedic Sports Medicine, Technical University of Munich, 81675 Munich, Germany; beitzelknut@tum.de; 3 Department of Orthopaedic Surgery, Juntendo University, Tokio 113-8421, Japan; 4 UNC Orthopaedics, University of North Carolina at Chapel Hill, Chapel Hill, NC 27599, USA; alex_creighton@med.unc.edu; 5 Southern Oregon Orthopedics, Medford, OR 97504, USA; pjdenard@gmail.com; 6 The Cleveland Shoulder Institute, Beachwood, OH 44194, USA; clevlandshoulder@gmail.com; 7 Department of Orthopaedic Sports Medicine, University of Arizona College of Medicine, Tucson, AZ 85006, USA; elederman1@icloud.com; 8 Dupage Medical Group, Elmhurst, IL 60126, USA; romeoortho@gmail.com; 9 Arthroscopy and Orthopedic Sportsmedicine, ATOS Orthoparc Clinic, 50858 Cologne, Germany

**Keywords:** reverse total shoulder arthroplasty, DSA, LSA, lateralization, distalization, radiographic analysis

## Abstract

Previous attempts to measure lateralization, distalization or inclination after reverse total shoulder arthroplasty (rTSA) and to correlate them with clinical outcomes have been made in the past years. However, this is considered to be too demanding and challenging for daily clinical practice. Additionally, the reported findings were obtained from heterogeneous rTSA cohorts using 145° and 155° designs and are limited in external validity. The purpose of this study was to investigate the prognostic preoperative and postoperative radiographic factors affecting clinical outcomes in patients following rTSA using a 135° prosthesis design. In a multi-center design, patients undergoing primary rTSA using a 135° design were included. Radiographic analysis included center of rotation (COR), acromiohumeral distance (AHD), lateral humeral offset (LHO), distalization shoulder angle (DSA), lateralization shoulder angle (LSA), critical shoulder angle (CSA), and glenoid and baseplate inclination. Radiographic measurements were correlated to clinical and functional outcomes, including the American Shoulder and Elbow Surgeons (ASES), Simple Shoulder Test (STT), Single Assessment Numeric Evaluation (SANE), and Visual Analogue Scale (VAS) score, active forward elevation (AFE), external rotation (AER), and abduction (AABD), at a minimum 2-year follow-up. There was a significant correlation between both DSA (*r* = 0.299; *p* = 0.020) and LSA (*r* = −0.276; *p* = 0.033) and the degree of AFE at final follow-up. However, no correlation between DSA (*r* = 0.133; *p* = 0.317) and LSA (*r* = −0.096; *p* = 0.471) and AER was observed. Postoperative AHD demonstrated a significant correlation with final AFE (*r* = 0.398; *p* = 0.002) and SST (*r* = 0.293; *p* = 0.025). Further, postoperative LHO showed a significant correlation with ASES (*r* = −0.281; *p* = 0.030) and LSA showed a significant correlation with ASES (*r* = −0.327; *p* = 0.011), SANE (*r* = −0.308, *p* = 0.012), SST (*r* = −0.410; *p* = 0.001), and VAS (*r* = 0.272; *p* = 0.034) at terminal follow-up. All other correlations were found to be non-significant (*p* > 0.05, respectively). Negligible correlations between pre- and postoperative radiographic measurements and clinical outcomes following primary rTSA using a 135° prosthesis design were demonstrated; however, these observations are of limited predictive value for outcomes following rTSA. Subsequently, there remains a debate regarding the ideal placement of the components during rTSA to most sufficiently restore active ROM while minimizing complications such as component loosening and scapular notching. Additionally, as the data from this study show, there is still a considerable lack of data in assessing radiographic prosthesis positioning in correlation to clinical outcomes. As such, the importance of radiographic measurements and their correlation with clinical and functional outcomes following rTSA may be limited.

## 1. Introduction

In the past years, the prevalence and clinical use of reverse total shoulder arthroplasty (rTSA) in the USA has dramatically increased by 40.8%, with 30,850 procedures being performed in 2013 compared to 21,916 in 2011 [1]. First designed by Paul Grammont in 1985 for the treatment of arthritic shoulders with severe cuff insufficiency [2,3], the rationale of rTSA was to medialize the center of rotation, and to distalize the humerus relative to the acromion, resulting in increased deltoid muscle tension in an attempt to facilitate active forward elevation (AFE) [4]. The initial design with a humeral inclination of 155° showed promising long-term functional outcomes, however it failed to restore active external rotation (AER) and led to significant scapular notching, which has been reported to occur in 74% to 88% of cases [2,3,5,6,7,8].

Thus, recent studies have focused on significant design modifications to improve active range of motion (ROM) by increasing lateralization on the glenoid side, implementing a more anatomic humeral inclination of 135°, and decreasing distalization of the humeral shaft [5,6,9,10,11,12]. As a result, Boileau et al. demonstrated that lateralization of the glenoid improved postoperative AER, and subsequently decreased the risk of scapular notching [5]. However, the increased use of rTSAs still elicits high rates of postoperative complications, occurring in 39% to 59% of cases [13,14]. However, of interest, Mahendraraj et al. recently showed that the distalization shoulder angle (DSA) and lateralization shoulder angle (LSA) may be reproducible measures, but seem to have only marginal correlation with postoperative clinical outcomes. As such, further investigations into the prognostic utility of minimally cumbersome rTSA measurement methodologies are warranted [15].

As intraoperative implant positioning has been shown to influence complication rates, attempts have been made to correlate pre- and postoperative radiographic measurements to clinical and functional outcomes [16,17]. However, these measurements and their correlation to outcomes in patients following rTSA are controversial among shoulder surgeons, while current evidence on the importance of these measurements is still lacking. Previous attempts to measure distalization of the humerus as well as medialization of the center of rotation have been considered to be too demanding for daily clinical practice [18,19], which has led Boutsiadis et al. to introduce more reproducible measurements and to evaluate their impact on postoperative clinical outcomes [20]. The authors showed that a lateralization shoulder angle (LSA) of 75° to 95° was correlated wirh increased AER, whereas a distalization shoulder angle (DSA) of 40° to 65° was correlated with increased AFE. However, the reported findings were obtained from a heterogeneous rTSA cohort using 145° and 155° designs and were limited in external validity and due to small sample sizes. Further, Jeon et al. found insufficient AFE in patients with increased postoperative lateral humeral offset (LHO) [21]. However, this was only observed when rTSAs were performed using medialized implants, to increase the force on the anterior deltoid (in patients with severe cuff tear arthropathy). As such, data on patients undergoing rTSA using a 135° prosthesis design remain limited.

The purpose of this study was to determine prognostic radiographic factors affecting clinical and functional outcomes in patients undergoing primary rTSA using a design with a humeral inclination of 135°. The authors hypothesized that there would be no significant correlation between radiographic measurements and clinical and functional outcomes following primary rTSA.

## 2. Methods

### 2.1. Study Design

A retrospective multi-center review was conducted on rTSA cases performed by 5 independent surgeons from 5 separate institutions between June 2013 and January 2018. Institutional review board permission was obtained prior to initiation of the study (IRB 17-202-2). Patients who underwent primary rTSA using an implant with a 135° humeral inclination for the treatment of cuff tear arthropathy or primary glenohumeral arthritis with a minimum follow-up of 2 years were included in the study. Patients were excluded if they underwent rTSA using a 155° prothesis design, were revision cases, had concomitant fractures of the humeral head or glenoid requiring surgery, or had neurovascular injuries.

### 2.2. Outcome Measures

American Shoulder and Elbow Surgeons (ASES), Simple Shoulder Test (SST), Single Assessment Numeric Evaluation (SANE) and Visual Analogue Scale (VAS) scores were collected preoperatively and at final follow-up [22,23]. Furthermore, range of motion, consisting of active forward elevation (AFE), active abduction (AABD) and active external rotation (AER), were recorded preoperatively and at final follow-up.

### 2.3. Surgical Procedure

All surgical procedures were performed utilizing a uniform implant design (Univers Reverse; Arthrex Inc., Naples, FL, USA). A deltopectoral approach was used in all cases. Subscapularis repair was based on surgeon preference. On the glenoid side, this system provides a 36, 39, or 42 mm glenosphere with neutral, 4 mm lateralized, or 2.5 mm inferior eccentric options. Inclination angle, glenosphere size and offset were based on intraoperative deltoid tension, implant stability, and notching according to surgeon’s preference. On the humeral side, the prosthesis has a modular cup which allows the surgeon to implant the component with either 135° or 155° of inclination. All humeral components were implanted by press-fitting. No patients required bone grafting.

### 2.4. Radiographic Evaluation

All patients had standard preoperative and postoperative radiographs (true anteriorposterior, y view and axillary view). Radiographic assessment was performed by two independent viewers blinded to patient outcomes. Radiographic measurements were performed on standard anteroposterior (AP) view performed at the most recent preoperative and last postoperative visit. Preoperative measurements included center of rotation (COR), critical shoulder angle (CSA) acromiohumeral distance (AHD), lateral humeral offset (LHO), and glenoid inclination (GI). Postoperative measurements included AHD, LHO, baseplate inclination (BI), distalization shoulder angle (DSA), and lateralization shoulder angle (LSA) [20,21,24,25,26].

**Figure 1:**
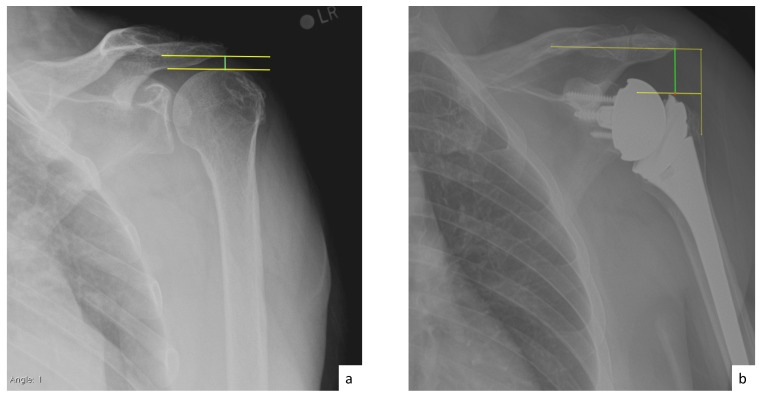
(**a**) Preoperative acromiohumeral distance (AHD; green line); (**b**) postoperative acromiohumeral distance (AHD; green line).

**Figure 2:**
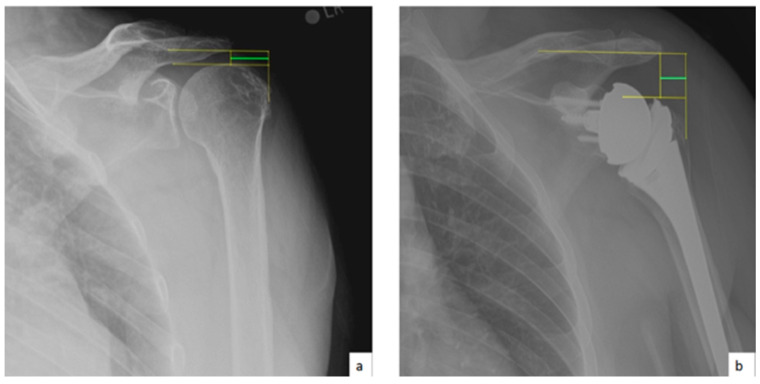
(**a**) Preoperative lateral humeral offset (LHO; green line); (**b**) postoperative lateral humeral offset (LHO; green line).

**Figure 3:**
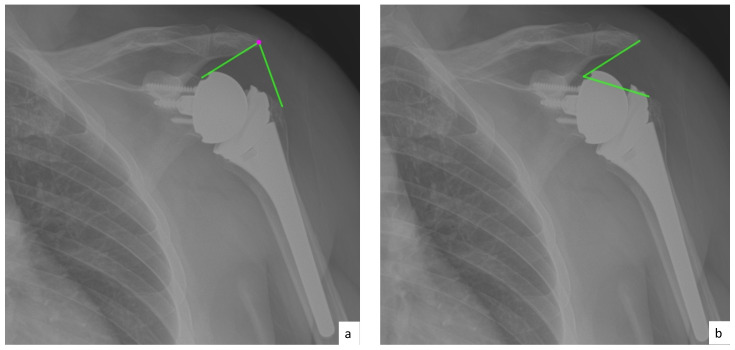
(**a**) Lateralization shoulder angle (LSA); (**b**) distalization shoulder angle (DSA).

**Figure 4:**
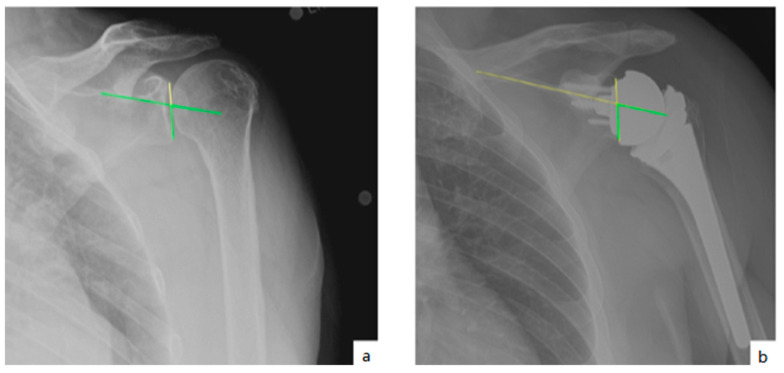
(**a**) Glenoid inclination (green angle); (**b**) baseplate inclination (green line).

**Figure 5:**
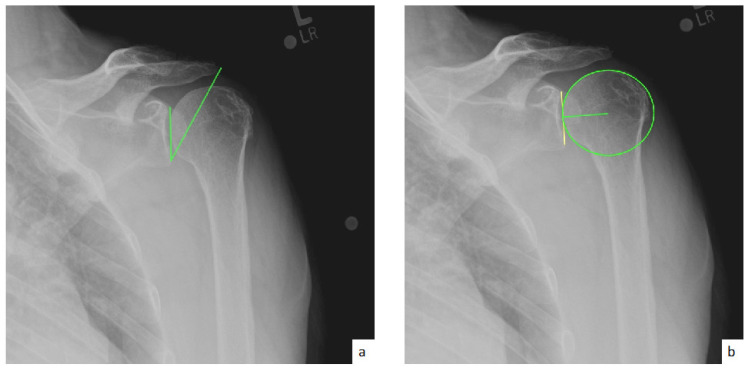
(**a**) Critical shoulder angle (CSA; green angle); (**b**) center of rotation (COI; green line).

AHD was measured by calculating the perpendicular distance between the most lateral portion of the undersurface of the acromion and a line parallel to the superior border of the greater tuberosity [21] (Figure 1). LHO was measured by determining the distance from the AHD line to the most lateral projection of the greater tuberosity [21] (Figure 2). LSA was measured by drawing a line from the superior glenoid tubercle to the most lateral border of the acromion and a second line from the most lateral border of the acromion to the most lateral border of the greater tuberosity. The angle between these two lines formed the LSA [20] (Figure 3a). DSA was measured by drawing a line between the most lateral border of the acromion and the superior glenoid tubercle and drawing a second line to connect the superior glenoid tubercle with the most superior border of the greater tuberosity. The angle between these two lines formed the DSA [20] (Figure 3b). Glenoid and baseplate were determined as the angle between the floor of the supraspinatus fossa and the glenoid fossa [25] (Figure 4). COR was measured by determining the best fit circle flush to the articular surface, identifying the center of the circle in the humeral head, and then measuring the distance of the perpendicular line between the center of the humeral head and the midpoint of the line connecting the superior and inferior glenoid tubercles [24] (Figure 5b). CSA was measured by a line from the superior pole to the inferior pole of the glenoid and a line from the inferior pole to the lateral edge of the acromion [26] (Figure 5a). In addition, scapular notching was graded according to the Nerot–Sirveaux classification and severity of preoperative cuff tear arthropathy was evaluated according to the Hamada classification [27,28].

### 2.5. Statistical Analysis

Descriptive statistics including mean and standard deviation for continuous variables and frequency and proportion for categorical variables were calculated to characterize the study groups. The relationships between clinical outcome measures and radiographic measurements were examined graphically with scatterplots and with Pearson correlation coefficients (rho). The effect of DSA and LSA on postoperative forward elevation was examined using a mixed effects linear model to account for patients nesting within surgeon’s practices. An interclass correlation coefficient (ICC) was calculated to determine reproducibility of the radiographic measurements. A *p*-value < 0.05 was considered statistically significant. All analyses were performed with Stata statistical software (StataCorp. 2017. Stata Statistical Software: Release 15. College Station, TX: StataCorp LLC).

## 3. Results

### 3.1. Subjects

**Figure 6:**
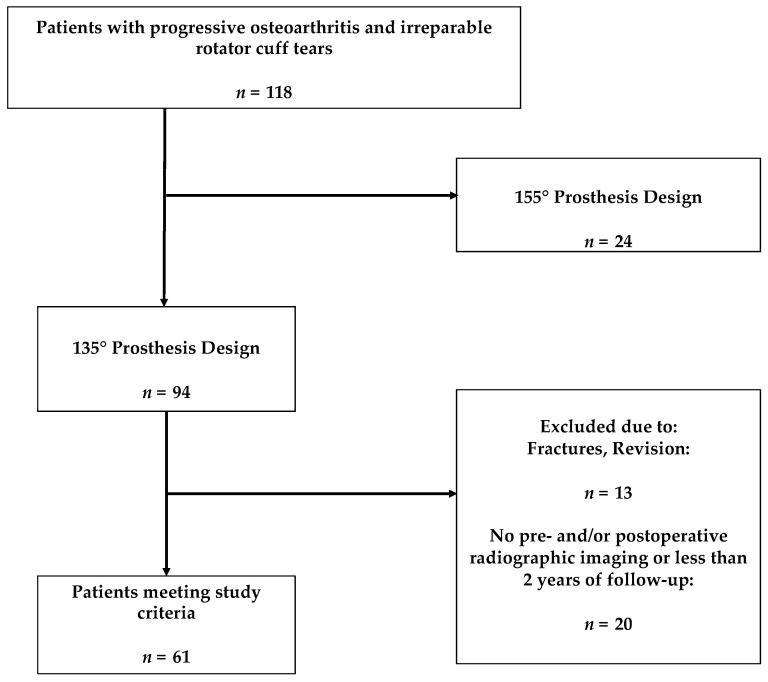
Flowchart displaying inclusion criteria.

Ninety-four rTSAs meeting the study criteria were performed during the study period. Of those, 61 were available at final follow-up (Figure 6). The mean age of patients was 69.2 ± 8.2 years (range: 53–88) with a mean follow-up of 3.1 ± 0.7 years (range: 2.0–4.2) years. Most patients were female (55.7%). Patient demographics are demonstrated in Table 1.

### 3.2. Clinical Outcome

Overall, there was significant improvement in all clinical outcome measures from pre- to postoperative. SST improved from 2.5 ± 1.8_pre_ to 8.0 ± 2.6_post_, SANE improved from 28.9 ± 22.7_pre_ to 80.7 ± 20.1_post,_ VAS improved from 6.0 ± 2.2 _pre_ to 1.4 ± 2.3_post_, ASES improved from 37 ± 14.5_pre_ to 78.1 ± 21.6_post_. At final follow-up, there was no significant difference in SST, SANE, VAS, and ASES when comparing patient populations of the different institutions.

In addition, there was significant improvement in ROM from pre-to postoperative. AFE improved from 92 ± 36°_pre_ to 131 ± 27°_post_, AABD improved from 69 ± 35°_pre_ to 109 ± 38°_post,_ AER improved from 29 ± 18°_pre_ to 42 ± 19°_post_ at final follow-up (*p* < 0.01, respectively). When comparing ROM of patients at the different institutions, no significant difference was found for AFE, AABD, or AER.

### 3.3. Inter-Rater Reliability of Radiographic Analysis

Inter-rater reliability was calculated for COR, Pre-CSA, Pre-AHD, Post-AHD, DSA, Pre-LHO, Post-LHO, LSA, glenoid inclination, baseplate inclination, Hamada and Notching grades. Reliability was found to be good for most of the radiographic measurements. However, Pre-AHD (ICC = 0.37; CI: 0.18–0.55) showed only poor reliability. Moderate to good ICC was found for COR (ICC = 0.68; CI: 0.51–0.8), DSA (ICC = 0.66; CI: 0.32–0.82) and glenoid inclination (ICC = 0.66; CI: 0.47–0.79). Mean values of radiographic measurements with corresponding inter-rater reliability are demonstrated in Table 2.

### 3.4. Correlation between Preoperative Radiographic Measurements and Clinical Outcomes

Pre-AHD was found to have a significant correlation with final AER (*p* = 0.016; *r* = 0.314). Additionally, Pre-LHO showed a significant correlation with final ASES (*p* = 0.032; *r* = −0.277). COR, CSA, and glenoid inclination had no significant influence on clinical outcomes at terminal follow-up (*p* > 0.05, respectively) (Appendix A Table A1).

### 3.5. Correlation between Lateralization and Clinical Outcomes

Post-LHO was found to significantly correlate with final ASES (*p* = 0.03; *r* = −0.281). Further, there was a significant correlation of LSA with final SST (*p* = 0.001; *r* = −0.41), final pain score (*p* = 0.034; *r* = 0.272), final SANE (*p* = 0.018, *r* = −0.308), and final ASES (*p* = 0.011; *r* = −0.327). Further, there was a significant correlation between LSA and final AFE (*p* = 0.033; *r* = −0.276). Correlations of LSA with final AER (*p* = 0.471; *r* = −0.096) and AABD (*p* = 0.824; r = 0.030) were found to be non-significant (Appendix A Table A1).

### 3.6. Correlation between Distalization and Clinical Outcomes

Post-AHD had a significant correlation with final SST (*p* = 0.025; *r* = 0.293). On the contrary, DSA showed no significant correlation to any clinical outcome measures. Post-AHD demonstrated a significant correlation to final AER (*p* = 0.002; *r* = 0.398). In addition, DSA significantly influenced final AFE (*p* = 0.02; *r* = 0.299). No significant correlations were found between DSA and final AER (*p* = 0.317; *r* = 0.133) and AABD (*p* = 0.283; *r* = 0.145).

### 3.7. Prediction of Active ROM

**Figure 7:**
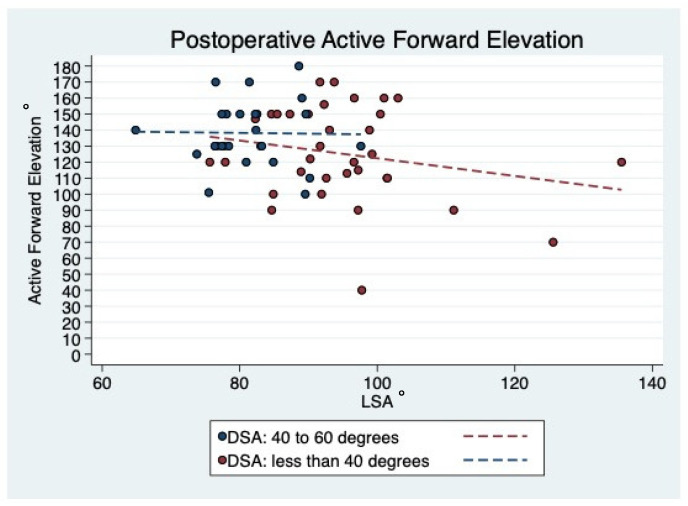
Scatterplot showing the linear correlation between DSA and LSA for final AFE. Good final AFE could be seen for DSA between 40 and 60° and LSA between 80 and 100°; Abbreviations: DSA = distalization shoulder angle; LSA = lateralization shoulder angle; AFE = active forward elevation.

**Figure 8:**
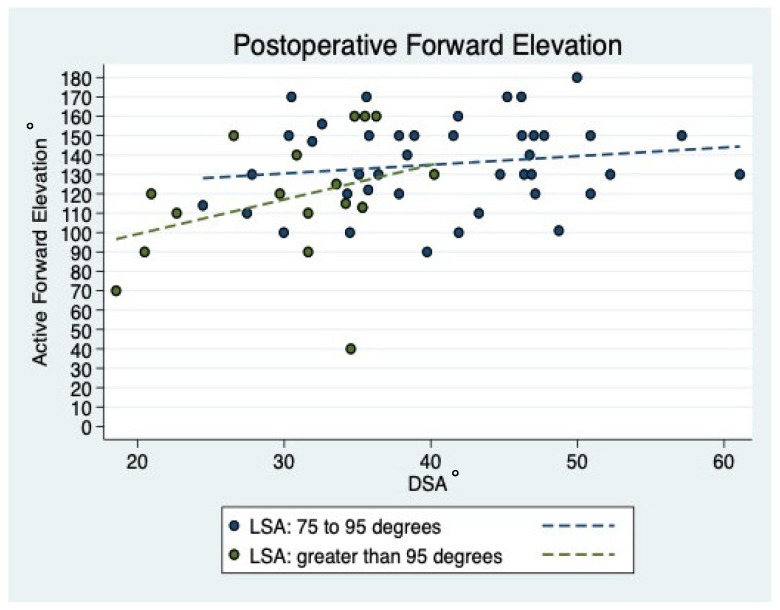
Scatterplot showing the linear correlation between LSA and DSA for final AFE. Good final AFE could be seen for DSA between 40 and 60° and LSA between 80 and 100°. Abbreviations: DSA = distalization shoulder angle; LSA = lateralization shoulder angle; AFE = active forward elevation.

The highest degree in AFE was observed in patients presenting with a postoperative DSA between 40° and 60°. Patients with an AFE < 100° (n = 5) were further shown to have a DSA smaller than 40° (Figure 7). When looking at the LSA, patients with an AFE < 100° (n = 4) had an LSA greater than 95°. The highest degree in AFE was observed in patients having an LSA between 75° to 95° (Figure 8). However, there was no statistically significant correlation between distalization (*p* = 0.317) and lateralization (*p* = 0.471) to AER at final follow-up.

## 4. Discussion

The most important finding of this study was that there was only a negligible correlation between radiographic measurements and clinical and functional outcomes following primary rTSA using a design with a humeral inclination of 135°. Even though statistically significant correlations between postoperative outcomes scores and radiographic measurements were found, these observations are of limited predictive value for outcomes following rTSA. The data gathered from this multi-center study indicate that the importance of radiographic measurements and their correlation with outcomes following rTSA may be limited.

In the postoperative setting, lateralization in rTSA can be expressed by different radiographic variables, including LHO, LSA, and COR. In their retrospective study, Jeon et al. demonstrated that an increased postoperative LHO was found to be a significant risk factor for poor restoration of postoperative AFE, when using an implant designed to be medialized [21]. In contrast, the data from this study showed that in a cohort using a lateralized implant, no significant relationship between preoperative and postoperative LHO and AFE could be demonstrated. However, in this study, post-LHO was found to significantly influence final ASES score, which may be of limited predictive value, as this finding did not allow for drawing a definite conclusion.

Increasing the lateralization of the COR in rTSA using a medialized implant design has been shown to result in greater active ROM [9]. As only few studies have focused on measuring COR in lateralized implants [18,29], the authors from this study could not find a significant relationship between COR, clinical outcomes, and final active ROM when using a lateralized rTSA design. Similar to a previous study by Boutsiadis et al. [20], who reported that patients achieved the highest degree in postoperative AFE and AABD with a DSA between 40° and 65° and the highest degree in AER when having a LSA of 75° to 95°, the findings of this study demonstrated a significant correlation between LSA and AFE as well as DSA and AFE. Additionally, the highest degree in AER was noted in patients having LSA values between 75° and 95°; however, a direct correlation of LSA and DSA with final AER and AABD could not be confirmed [20]. This may be explained by existing differences in implant designs being used, as all included patients uniformly underwent rTSA using a lateralized design with a humeral inclination of 135°. In contrast, Boutsiadis et al. included patients with two different implant designs (145° and 155° humeral inclination) [20].

Additionally, a positive correlation between LSA and DSA could be shown, which is consistent with the current literature [20]. In a lateralized rTSA design, a lower LSA, which corresponds to a more medialized implant, is associated with a larger DSA, indicating a greater distance between the acromion and humerus. To this, the findings from this study suggest that a LSA greater than 100° correlates with a DSA of less than 40°, thus reducing final AFE. Considering the current literature, lateralization of rTSA has been shown to increase postoperative AFE and AER by restoring the anatomic COR, while optimizing recruitment of the muscle fibers [6,30,31]. However, LSA was noted to be between 75° and 95° for optimal implant lateralization, with excessive lateralization resulting in less active ROM.

Increasing distalization, in order to improve tension on the deltoid muscle, has been shown to play an important role in rTSA [4]. In a computer-based model using different humeral offset and stem designs, Lädermann et al. demonstrated a strong positive linear relationship between AHD and AFE and AABD [6]. Furthermore, the authors showed that AHD decreased by 6 mm when switching from a 155° inlay design to a 135° onlay design. Even though it was shown that a higher AHD, expressed as arm lengthening, was related to a higher degree in AFE [6], the exact amount of arm lengthening remains inconclusive [6,18,32,33].

First introduced by Moor et al. [26], the CSA has been reported to be a reproducible radiographic index. As a larger CSA has been found to be associated with degenerative rotator cuff tears, there is still limited knowledge regarding its influence on rTSA [34]. Even though Roberson et al. [34] reported improved AFE in patients with a lower CSA, no significant relationship between CSA and clinical outcomes scores or final active ROM was found in this study.

Taking these findings into account, there remains a debate regarding the ideal placement of the components during rTSA to most sufficiently restore active ROM while minimizing complications such as component loosening and scapular notching. Additionally, as this study further verified, there is still a considerable lack of data in assessing radiographic prosthesis positioning in correlation with clinical outcomes. This may lead shoulder surgeons to overestimate current data and the importance of radiographic measurements and their correlation with outcomes following rTSA.

There are several limitations to the study. First, the study cohort was not randomized. Second, although outcomes were collected prospectively, data were reviewed retrospectively, which could create selection bias. Third, all radiographic measurements are highly dependent on patient orientation during radiographic imaging, as angles and distances are influenced by the position of the scapula as well as rotation of the humerus. However, this reflects daily clinical practice, as radiographic imaging, even if standardized, can show significant variances. Fourth, the multi-center design of this study including five surgeons from different sites leads to differences in implant positioning and intraoperative and postoperative outcomes. However, for the purpose of this study, the authors intended to demonstrate that even with high experience and expertise in this field, the observations and findings from this study and its subsequent comparison to the current literature should be interpreted with careful attention.

## 5. Conclusions

Negligible correlations between pre- and postoperative radiographic measurements and clinical outcomes following primary rTSA using a 135° prosthesis design were demonstrated. However, these observations are of limited predictive value for outcomes following rTSA. Subsequently, there remains a debate regarding the ideal placement of the components during rTSA to most sufficiently restore active ROM while minimizing complications such as component loosening and scapular notching. Additionally, as the data from this study showed, there is still a considerable lack of data in assessing radiographic prosthesis positioning in correlation to clinical outcomes. As such, the importance of radiographic measurements and their correlation with clinical and functional outcomes following rTSA may be limited.

## Figures and Tables

**Table 1:** Patient demographics (*N* = 61).

	*n*	%
Sex		
Male	27	44.3
Female	34	55.7
Mean age ± SD (years)	69.2 ± 8.2	
Mean follow-up ± SD (years)	3.1 ± 0.7	
Dominant Arm Involved	32	52.0
Right Shoulders	33	54.0
BMI	29.9 ± 7.0	

**Table 2:** Inter-rater reliability for all radiographic measurements and mean values for radiographic analysis.

	Mean ± SD	ICC	ICC 95% CI	Reliability
COR	20.9 ± 3.9 mm	0.68	[0.51, 0.8]	Moderate-Good
Pre CSA	35.2 ± 4.5 deg	0.9	[0.9, 0.94]	Good
Pre AHD	5.1 ± 3.2 mm	0.37	[0.18, 0.55]	Poor
Post AHD	26.3 ± 9.5 mm	0.88	[0.82, 0.93]	Good
DSA	38.6 ± 9.6 deg	0.66	[0.32, 0.82]	Moderate-Good
Pre LHO	9.9 ± 5.7 mm	0.86	[0.79, 0.91]	Good
Post LHO	9.5 ± 6 mm	0.84	[0.75, 0.89]	Good
LSA	89.2 ± 11.9 deg	0.84	[0.73, 0.9]	Good
Glenoid inclination	81.2 ± 6.8 deg	0.66	[0.47, 0.79]	Moderate-Good
Baseplate inclination	83.2 ± 6.4 deg	0.79	[0.69, 0.86]	Good

Abbreviation: COR = center of rotation; Pre CSA = preoperative critical shoulder angle; Pre AHD = preoperative acromiohumeral distance; Post AHD = postoperative acromiohumeral distance; DSA = distalization shoulder angle; Pre LHO = preoperative lateral humeral offset; Post LHO = postoperative lateral humeral; LSA = lateralization shoulder angle.

## Data Availability

The data presented in this study are available on request from the corresponding author. The data are not publicly available due to ethical reasons.

## Appendix A

**Table A1:** Table showing correlation between radiographic analysis and final clinical outcome scores.

		ASES	SANE	SST	VAS	FE	ABD	ER
**COR**	r	−0.079	0.080	0.006	0.154	0.170	−0.176	−0.011
	*p*−value	0.547	0.545	0.966	0.235	0.194	0.190	0.937
**Pre CSA**	r	−0.035	0.056	−0.056	0.051	−0.211	−0.212	−0.187
	*p*−value	0.794	0.672	0.677	0.697	0.105	0.113	0.156
**Pre AHD**	r	0.085	0.142	0.124	−0.059	−0.051	−0.015	0.314
	*p*−value	0.518	0.284	0.349	0.652	0.697	0.910	**0.016**
**Pre LHO**	r	−0.277	−0.243	−0.251	0.177	−0.048	−0.037	0.035
	*p*−value	**0.032**	0.064	0.055	0.173	0.716	0.783	0.790
**Post−AHD**	r	0.150	0.179	0.293	−0.135	0.398	0.111	0.233
	*p*−value	0.253	0.174	**0.025**	0.299	**0.002**	0.411	0.075
**Post LHO**	r	−0.281	−0.215	−0.197	0.193	0.086	0.045	−0.003
	*p*−value	**0.030**	0.102	0.135	0.136	0.513	0.739	0.985
**DSA**	r	0.169	0.099	0.234	−0.145	0.299	0.145	0.133
	*p*−value	0.198	0.456	0.075	0.266	**0.020**	0.283	0.317
**LSA**	r	−0.327	−0.308	−0.410	0.272	−0.276	0.030	−0.096
	*p*−value	**0.011**	**0.012**	**0.001**	**0.034**	**0.033**	0.824	0.471
**Inclination Glenoind.**	r	−0.066	−0.156	−0.095	0.132	0.072	0.176	0.104
	*p*−value	0.614	0.238	0.473	0.310	0.583	0.191	0.435
**Inclination Baseplate**	r	−0.121	0.038	−0.102	0.123	0.122	0.106	0.050
	*p*−value	0.356	0.776	0.442	0.347	0.353	0.433	0.710
**Hamada**	r	−0.009	0.067	−0.031	0.013	0.156	−0.141	−0.289
	*p*−value	0.947	0.613	0.817	0.919	0.233	0.297	0.026
**Notching**	r	−0.030	−0.165	0.042	0.151	−0.246	−0.133	−0.214
	*p*−value	0.818	0.213	0.754	0.244	0.058	0.325	0.104

Significant values (*p* < 0.05) are highlighted. Abbreviation: COR = center of rotation; Pre CSA = preoperative critical shoulder angle; Pre AHD = preoperative acromiohumeral distance; Post AHD = postoperative acromiohumeral distance; DSA = distalization shoulder angle; Pre LHO = preoperative lateral humeral offset; Post LHO = postoperative lateral humeral; LSA = lateralization shoulder angle; FE = postoperative forward elevation; ABD = postoperative abduction; ER = postoperative external rotation; ASES = Postoperative American Shoulder and Elbow Surgeons; SST = Postoperative Simple Shoulder Test; VAS = Postoperative Visual Analogue Scale; SANE = Postoperative Single Assessment Numeric Evaluation.
